# Perspectives of Patients, Health Care Professionals, and Developers Toward Blockchain-Based Health Information Exchange: Qualitative Study

**DOI:** 10.2196/18582

**Published:** 2020-11-13

**Authors:** Keehyuck Lee, Kahyun Lim, Se Young Jung, Hyerim Ji, Kyungpyo Hong, Hee Hwang, Ho-Young Lee

**Affiliations:** 1 Office of eHealth Research and Business Seoul National University Bundang Hospital Seongnam-si Republic of Korea; 2 Department of Family Medicine Seoul National University Bundang Hospital Seongnam-si Republic of Korea; 3 Department of Nuclear Medicine Seoul National University Bundang Hospital Seongnam-si Republic of Korea

**Keywords:** blockchain, health information exchange, qualitative study

## Abstract

**Background:**

Although the electronic health record system adoption rate has reached 96% in the United States, implementation and usage of health information exchange (HIE) is still lagging behind. Blockchain has come into the spotlight as a technology to solve this problem. However, there have been no studies assessing the perspectives of different stakeholders regarding blockchain-based patient-centered HIE.

**Objective:**

The objective of this study was to analyze the awareness among patients, health care professionals, and information technology developers toward blockchain-based HIE, and compare their different perspectives related to the platform using a qualitative research methodology.

**Methods:**

In this qualitative study, we applied grounded theory and the Promoting Action on Research Implementation in the Health Service (PARiHS) framework. We interviewed 7 patients, 7 physicians, and 7 developers, for a total of 21 interviewees.

**Results:**

Regarding the leakage of health information, the patient group did not have concerns in contrast to the physician and developer groups. Physicians were particularly concerned about the fact that errors in the data cannot be easily fixed due to the nature of blockchain technology. Patients were not against the idea of providing information for clinical trials or research institutions. They wished to be provided with the results of clinical research rather than being compensated for providing data. The developers emphasized that blockchain must be technically mature before it can be applied to the health care scene, and standards of medical information to be exchanged must first be established.

**Conclusions:**

The three groups’ perceptions of blockchain were generally positive about the idea of patients having the control of sharing their own health information. However, they were skeptical about the cooperation among various institutions and implementation for data standardization in the establishment process, in addition to how the service will be employed in practice. Taking these factors into consideration during planning, development, and operation of a platform will contribute to establishing practical treatment plans and tracking in a more convenient manner for both patients and physicians. Furthermore, it will help expand the related research and health management industry based on blockchain.

## Introduction

### Background

The Health Information Technology for Economic and Clinical Health (HITECH) Act was enacted in 2009 in the United States following evidence that the use of electronic health records (EHRs) can improve the quality of health care [1]. Since the law came into effect, the health care industry has seen a number of changes [2]. The EHR adoption rate has increased by 8% annually, which reached 96% of general medicine and surgical hospitals, 89% of rehabilitation hospitals, and 87% of children’s hospitals in 2017 [3,4]. An EHR adoption program can achieve full success through health information exchange (HIE) because, similar to the exchange of information over the internet, medical information has the greatest value when exchange occurs without barriers [5]. However, according to statistics from the US government, significant progress must be made to reach a fully networked, patient-centered, integrated HIE system [6]. The situation in Korea is not much different. The government of the Republic of Korea has been developing an HIE system since 2006, but it is not yet fully operational because the information shared is only limited to medical institutions and not all data related to health care consumers’ needs are provided [7-10].

Recently, blockchain has come into the spotlight as a technology to solve this problem [11-15]. Blockchain technology is a distributed ledger system that has no central authority, and the metadata are stored in “blocks,” also known as on-chain data, which are securely created and are immutable owing to the use of unique cryptography [16]. Metadata on the blocks are linked to off-chain data in local hospital servers. Unlike traditional methods of storing and recording data from central servers, with blockchain technology the users validate, record, and manage the same data by themselves via person-to-person networks to ensure the trustworthiness, transparency, and security of the data. Owing to these benefits, blockchain technology is being adopted in various industries to foster innovation, and the health care industry is no exception.

A previous review paper explored the current status of health care worldwide, and indicated that proposed solutions impacted by blockchain are mostly EHRs, personal health records (PHRs), and clinical trial support systems [17]. The processes within the target systems were mainly focused on storage, sharing, and the exchange and access of health care data. However, uptake of the health care blockchain has been slow around the world [18]. The main reason is users’ lack of motivation to use the blockchain platform actively. The lack of motivation in health care blockchain is mostly due to not recognizing its advantages such as the improvement to data security, privacy, integrity, interoperability, and authentication [19]. In terms of HIE, the advantage of this technology is that the individual has the authority to control the scope of the institution and information, while ensuring security. The disadvantage of blockchain is that the data are verified by many parties and the capacity of the block is limited, resulting in a slow and difficult task to process large volumes [20,21]. In addition, blockchain is known to be inflexible because once the information is recorded on the block, it is very hard to delete and there is almost no way to determine whether the private key for personal authentication and decryption has been hacked or lost [22].

However, in a health care blockchain, patients’ private keys can be stored in hospitals with well-established security systems and there are a few ways in which the above-mentioned disadvantages can be overcome. First, with only limited trusted parties such as hospitals and government institutions involved in the blockchain consensus algorithm by using a private blockchain, the verification process can be quicker [19]. Second, data that are too sensitive or large can be managed externally via the off-chain method, which resolves the inflexibility and difficulty in deleting data from the blockchain while increasing the efficiency in processing data of smaller size. In addition, in terms of HIE, there is a drawback due to the open nature of the blockchain, as patient information can be exposed to other parties not authorized by the patients [23]. This can be solved through the use of smart contracts in blockchain with dynamic consent, which allows the individual with the authority to control the parties that can access the information and to what extent, while ensuring security and data integrity [24].

### Previous Research

A previous study revealed that HIE can reduce unnecessary medical expenses [8]. Despite the advantage of HIE, the adoption rates have still been low [25,26]. One study analyzed and reported the perceptions of medical staff on the health care blockchain [27]. Another study analyzed how to integrate data from mobile health care apps into the blockchain [28]. In addition, research has focused on how to manage PHR data in a blockchain [29], and how blockchain technology can be used to recruit patients and manage the entire process for clinical trials [30,31]. However, no studies have proven the effectiveness of applying blockchain technology directly to HIE, and in particular, there has been no research on how consumers, patients, or developers appreciate such blockchain-based medical information exchange based on a qualitative research method. Therefore, the aim of this study was to establish the grounds for developing a blockchain platform that can be utilized in the actual health care environment by analyzing the awareness and concerns of the stakeholders about blockchain health care based on qualitative research methodology.

### Aim

Regarding the limitations in previous studies, the objective of this study was to analyze the awareness among patients, health care professionals, and information technology (IT) developers toward blockchain-based HIE, and to compare their different perspectives on the platform using qualitative research methodology. The results of this study can serve as the grounds for developing a blockchain platform that can be utilized in the actual health care environment.

## Methods

### Participants

This study was designed using a qualitative approach to analyze the awareness among various stakeholders toward sharing personal health information based on blockchain technology that is patient-centered. The participants included patients (medical service consumers), physicians (health care providers), and technical developers. Semistructured interviews were conducted to collect and analyze their awareness and views of health care blockchain. No other specific inclusion criteria were set when selecting the participants. However, only the developers who had experience in developing health management systems, hospital information systems, or information exchange systems were selected to collect data to meet the purpose of the study.

### Design

We applied grounded theory methodology, which is used when a conceptual framework of a phenomenon has not been defined clearly, understanding of the relationship between concepts is lacking, or when relevant and irrelevant variables cannot be determined because studies have not been conducted repeatedly on a certain topic [32]. Grounded theory uses inductive reasoning alongside qualitative data collection. In this study, we recorded the opinions of three types of stakeholders on blockchain-based HIE through an iterative process of discussion based on grounded theory. In addition, the interview questions were determined based on the Promoting Action on Research Implementation in the Health Service (PARiHS) framework [33], which was developed to provide insight on how the topics discussed in a study are implemented in the actual environment (ie, how to apply the knowledge into practice). This is largely based on three factors: literature or knowledge-based evidence (Evidence), consideration of situations (Context), and facilitating elements to introduce research details into practice (Facilitation) [34]. Since the broader aim of this study was to preliminarily develop a PHR system that incorporates blockchain-based HIE, we adopted the PARiHS framework to be able to transfer the results to the development of a PHR.

Based on this theory and framework, semistructured interview questionnaires were developed for each interview group. In this study, Evidence was subdivided into Awareness and Prior Experience; Context was subdivided into Existing Problems and Attitudes; and Facilitation was subdivided into Perceived Risk, Perceived Benefits, and Suggestions. Several questions were assigned to each subfactor. The list of detailed questions is shown in Multimedia Appendix 1. The interviewers explained the basic concept of blockchain during the interview process so that the participants could respond to the research questions with proper background knowledge. This study also followed the COREQ (Consolidated Criteria for Reporting Qualitative Research) guidelines [35].

### Setting

The interviewed patients and physicians were recruited from Seoul National University Bundang Hospital, which has supervised a government-led pilot project on building an HIE system; thus, the health care professionals’ understanding of IT and information exchange based on an IT system is fairly high at this hospital [10,36-39]. Furthermore, various IT infrastructures are provided at this hospital, such as guidance or a payment kiosk for patients, smart bedside stations, patient portals, and patient guide systems [40-48]. Therefore, the patients are familiar with the IT infrastructures at the hospital, and the researchers decided that it would be easy to help the patients and health care professionals at this hospital clearly understand the concept of consumer-centered HIE based on blockchain technology.

For participant recruitment, purposive sampling and snowball sampling were adopted together. Purposive sampling was used for recruiting patients and physicians, whereas snowball sampling was used for recruitment of IT developers. Purposive sampling is a nonprobability sampling method in which researchers select a sample in accordance with their own judgment [49]. Snowball sampling is also a nonprobability sampling method in which the samples are recruited from the pool of participants by continuously being introduced to more potential study participants. This method is useful when it is difficult to find study participants who belong to a certain population [50]. Based on a previous study, interviews were designed to include at least 5 people per group [35].

We included 7 patients, physicians, and developers, respectively, for a total of 21 interviewees, and data saturation was confirmed while conducting the interviews. When selecting the participants, patients with chronic illness who visited the hospital often were selected primarily because they were already familiar with hospital processes. Furthermore, developers who had experience in developing PHR systems, hospital information systems, or information exchange systems were selected to collect data that met the purpose of the study. Physicians who were professors working at Seoul National University Bundang Hospital for at least 1 year and had experience in HIE were selected as interviewees.

### Data Collection

Two researchers conducted face-to-face, semistructured interviews. One researcher who was working on a pilot project in developing nationwide HIE in South Korea led the interviews in the physician and developer groups. She is also a trained data scientist and health information analyst. The positive relationship with the participants in the two groups created an open atmosphere by encouraging participants to speak more freely about their experience. The other researcher led the interviews in the patients group. The interviews were recorded with the participants’ consent and lasted about 20 to 40 minutes. There were no other individuals present except for the participants and interviewers. All participants signed the consent form voluntarily and were informed that they could freely withdraw from the study at any time. During the sessions, they referenced the semistructured interview questionnaire that covered all topics relevant to their experiences and opinions regarding a blockchain-based PHR. All sessions were conducted in a one-on-one manner.

The researchers followed the interview guidelines developed based on previous studies and were approved by members of the eHealth research team at Seoul National University Bundang Hospital.

### Data Analysis

Explorative content analysis was performed to capture the opinions and perspectives on blockchain-based HIE through a PHR. The recorded interviews were transcribed by two researchers who had been researching blockchain technology for more than 1 year, who crosschecked each other’s work to produce the final transcript for analysis. Subsequently, each of the two researchers reviewed the transcription and then coded each subfactor. The coding manual was defined while inspecting the domain system together during categorization. The coding schemes developed by each researcher independently were then compared and analyzed according to the final coding manual, and the results were drawn after reviewing and agreeing upon the interview details.

In addition, the transcripts were further reviewed and corrected repeatedly by all coresearchers to enhance the accuracy of transcriptions. All researchers verified the results until reaching a consensus on clarified classifications.

### Ethics

This study was approved by the Institutional Review Board of Human Research of Seoul National University Bundang Hospital, Republic of Korea (Protocol No. B-1910-571-302).

## Results

### Participants

The average age of the interviewees was 51 years (SD 15.7 years, range 15-72 years). Considering that the majority of patients at the hospital are middle-aged and elderly, middle-aged people were preferentially recruited since they are more IT-savvy. The demographics of the study participants are presented in Table 1.

**Table 1:** Demographic characteristics of participants (N=21).

Characteristic		n (%)
**Interviewee Group**		
	Patients	7 (33)
	Doctors	7 (33)
	Developers	7 (33)
**Gender**		
	Male	16 (76)
	Female	5 (24)
**Age (years)**		
	<30	0 (0)
	30-39	4 (19)
	40-49	11 (52)
	50-59	5 (24)
	60-69	0 (0)
	≥70	1 (5)
**Length of career (years)^a^**		
	1-4	1 (7)
	5-10	4 (29)
	11-15	5 (36)
	16-20	3 (21)
	≥21	1 (7)

^a^Doctors and developers only (N=14).

### Evidence: Awareness of Blockchain, HIE, and PHR

The detailed responses to the questions for all three stakeholder groups are presented in Multimedia Appendix 2, and an overview of the responses is provided in Table 2. Most patients who were interviewed (5/7) did not know about PHR and HIE. In addition, none of the patients was aware of blockchain-based HIE. By contrast, all physicians and developers were aware of, had knowledge about the usage, or participated in the development of PHR, HIE, and health care blockchain.

**Table 2:** Summary of the interviews for each component of the Promoting Action on Research Implementation in the Health Service (PARiHS) framework.

PARiHS Component		Patients		Physicians		IT^a^ developers	
**Evidence**							
	Awareness of health blockchain and the concepts of HIE^b^ and PHR^c^		No		Yes		Yes
	Prior experience with HIE		Yes, sharing information (self, parents, children)		Yes, sharing patient information		Yes, developed a related service
**Context**							
	Existing problems in exchanging health information		Was not guided by the hospital		Too much unnecessary information; not all patients/hospitals participate		No problem in the system; the utilization is low
	Attitudes about blockchain-based patient-centered HIE		Positive		Somewhat positive		Somewhat positive
**Facilitation**							
	Perceived Risk		Difficult to use		Concerned about security issue; possibility of legal conflicts if all information is shared. Difficult to edit data. Health care professionals would become more conservative during treatment		Possibility of data loss due to users’ inexperience. Data standardization needs to be done at each institution/corporation
	Perceived Risk (information safety)		No		Yes		Yes
**Suggestion**							
	Function		Guardian controlling information on behalf of a patient (eg, elderly parent, underage children). Information sharing network for rare blood type/diseases		Recruiting patients for clinical trials, receiving only the requested information		Distributed storage of actual data, setting managing entity for metadata
	Utilization measures		Ease of use, education for usage, and recommendation by health care professionals		Government leadership at initial stabilization stage (incentive system, etc)		None
Data exchanging institutions			Hospital: Yes; Clinical Research Institute: Yes; Corporation: No; Health Management Center: indeterminate		Hospital: Yes; Clinical Research Institute: Yes; Corporation: indeterminate; Health Management Center: indeterminate		Hospital: Yes; Clinical Research Institute: Yes; Corporation: Yes; Health Management Center: Yes

^a^IT: information technology.

^b^HIE: hospital information exchange.

^c^PHR: personal health record.

All participants in the patients group had experience in sharing personal health information by submitting their health information for hospital, insurance claim, and health management purposes. Several participants also had experience in sharing health information of their parents or children. All participants reported that they physically visited the hospital to receive necessary documents. All patients mentioned that this was a complicated, time-consuming, and burdensome process. Nevertheless, the patients followed the process because it is a health-related issue, required for an insurance claim (ie, economical), and to avoid repeated medical checkups. Most of all, the patients reported that the main reason for selecting this route was because they were “told by the hospital to do so.”

Physicians responded that they understood HIE and PHR in theory but did not frequently use such a system. Some IT developers responded that they did not have difficulty in developing the HIE or PHR services but faced difficulty in data mapping during the planning stage as they lacked understanding of the business (Table 3).

**Table 3:** Prior experience related to the awareness of blockchain, hospital information exchange (HIE), and personal health record (PHR) systems.

Group	Quotes
Patients	“My parents who live in Gangneung were ill, therefore they needed to be taken care of by the family. So, my parents and I transferred the records from a hospital in Gangneung to Bundang [where the participant lives]. I submitted the health records when making an insurance claim. I had to go to the hospital in Gangneung where my parents used to attend just to get the documents I needed. I submitted the documents for the insurance company through fax. It was too far, so I had to take a day off from work. It was too complicated and inconvenient. I had to see the doctor again to get issued documents, and could not get all the necessary documents from one place. I would have not done this if it was not for my parents’ health.” “After visiting a local gynecologic clinic, I had to go to a bigger hospital where I submitted examination results and referral notes. I had to submit health records when making an insurance claim. It’s fine when I’m not busy, but it is a burden to get all these different kinds of documents when I’m busy. It would be better if they can give me all the documents at once, otherwise it’s very inconvenient.”
Physicians	“I’m aware of such a service, and I sometimes look up patient information on a health information exchange system. But I don’t use it often.” “Not all hospitals participate, and it didn’t seem like the information I need is organized in a way so that I can easily find it. I don’t use it often because I have many patients to checkup, so I don’t want to waste time.” “I find it quite useful. If the patient is referred from another hospital, it was good for checking their diagnosis and examination result.”
Developers	“I was involved in the development process. Aside from the development itself, data mapping can proceed only if we understand the business. So, the hardest part was to discuss this with busy doctors and to make them understand it fully.”

### Context: Existing Problems in Exchanging Health Information

All patients had a positive response to PHR and HIE after it was explained to them. They responded that they had not been able to use such a system because they were not informed of a more convenient way. Although all participants use smartphones, the overall IT literacy is not high. Hence, they reported that looking up information on the internet and learning new methods by themselves is difficult. One patient who was aware of the PHR app and HIE service responded that the PHR app is merely used for checking information and the HIE service provided the information-sharing service between hospitals, and is not very useful since he does not visit any other hospital often. For physicians, the HIE system does not contain information that is actually needed, and an excessive amount of information is randomly provided and is thus not being used often during treatment. Further, patient information could not be found in some cases because not all hospitals were participating in the system. The developers responded that the system is well established although its utilization is low (Table 4).

**Table 4:** Opinions about existing problems in hospital information exchange (HIE).

Group	Quotes
Patients	“I was told to do so. Administrative procedures at the hospital seem pretty complicated, so I just did what they told me to do without any doubts. I didn’t know about such a great system [HIE service] because no one told me. I would have used it if I knew…” “I really needed a service like this. I visit the hospital often, and so do the elderly in my family… But, do I need to go to the hospital to use it?” “I’ve heard of the health information exchange service, but I don’t feel the need to use it because I don't switch hospitals often unless the information is shared somewhere else.”
Physicians	“There is too much unnecessary information. The time is limited, and I get confused because some of the information is not what I need for treatment. It would be great if the exam results are related to the actual referral be highlighted.” “In ophthalmology, blood test results in the system are not very useful for us. The referral written on paper actually has more relevant information.” “Because not all hospitals participate in the system, sometimes patient information cannot be searched. Also, it’s difficult to find out the situation of the institution I’m referring the patient to. For example, I’d like to refer a patient to get an abdominal ultrasound after 6 months, but I can’t tell if that institution can perform abdominal ultrasound. When I’m referring a patient to several departments, I’m not sure if I need to fill out the information for each department separately or do it as a whole.”
Developers	“No improvement is necessary in terms of the system. The biggest problem is that its utilization is low. Legal or systematic actions should be taken to standardize data exchange and make it mandatory.” “It’s not being used widely after development. Various cases need to be discovered to make advancements for higher effectiveness. I’m disappointed that the usage rate is so low.”

### Context: Attitudes on Blockchain-Based Patient-Centered HIE

Patients were positive about the idea of sharing personal information not only with hospitals but also with insurance companies or health management service providers under their control. Many patients receive diverse treatments and manage their health in addition to visiting hospitals. For example, some patients with lower back pain visited orthopedics and traditional medicine clinics, and engaged in personal training exercises at the same time, in which they provided X-ray results or diagnostic information to traditional medicine doctors and personal trainers. They were particularly positive about how they could provide any information to these institutions on their own will using smartphones.

Physicians were also very positive about the idea of requesting and receiving only the information they or their patients need, unlike the existing HIE service in which data formatted in a set Clinical Document Architecture format must be sent or received. Developers identified the purpose of such a system or service but pointed out that the data managed independently by various institutions and companies need to be standardized to enable searching of and sharing certain data using blockchain, ultimately implementing interoperability in performing actual tasks. However, they responded that the most challenging part would be having institutions and companies voluntarily follow these processes and participate in the system. Once this issue is resolved, they explained that there will be no technical difficulty in creating blockchain-based user authentication or information exchange systems (Table 5).

**Table 5:** Attitudes on blockchain-based patient-centered hospital information exchange (HIE).

Group	Quotes
Patients	“So now I can just use my phone to send my information to wherever I want without having to visit the hospital after going through the consent process. It’s less work for everyone. Not just for hospitals but it’s easier to send documents to insurance companies as well.” “I think it’s great. If I happen to end up at an emergency room, the doctors would know what kind of illness or allergies I have?” “Because of my lower back pain, I visit an orthopedic doctor but also a traditional medicine clinic near where I live. The doctor at the traditional medicine clinic asked for my X-ray when I visited. Now I can show him my X-ray without having to visit the hospital I usually go to?”
Physicians	“I think that the most ideal way is to exchange patient-centered medical information through blockchain.” “I would like to participate in the blockchain-based medical information exchange platform, but I suggest that the information be standardized before that.”
Developers	“Blockchain is expected to be used a lot in the health care fields. Right now, the PHR [personal health record] only has a simple prescription record and a diagnostic record, but I think it would be helpful for you at the enterprise level if you could obtain a lot of data related to health care.”

### Facilitation: Perceived Risk

Patients were very positive about the blockchain-based patient-centered HIE; however, they were reluctant or even afraid to search for new information and learn new methods as blockchain is a relatively new technology. Among the 7 patients who were interviewed, 6 patients responded that they did not try to learn new IT. However, they were willing to learn and use the new system if their primary care physician or the hospital provided instructions and education. They were not particularly concerned with a leakage of health information, unlike physicians, who were sensitive to information security issues.

There are cases of errors made in patient charts, and physicians were concerned with being unable to revise or edit information in blockchain. If all information can be accessed by anyone upon patient consent, the information could be shared with lawyers at any time, which would create an environment in which legal conflicts regarding medical practices may occur more frequently. The physicians explained that the system would cause them to be more conservative during treatment. Some also emphasized that it would encourage doctor shopping even more. Developers also considered security as the greatest risk. Specifically, the developers thought that if information gathered on the blockchain platform was hacked or released accidentally, all medical information could be exposed. They also expressed concern about the situation in which users could not access their medical information at all if they inadvertently lost their private keys. The developers responded that blockchain has been proven only theoretically so far, and its reliability of information security in the real world has not been verified sufficiently through experiences (Table 6).

**Table 6:** Perceived risks of a blockchain-based hospital information exchange (HIE) system.

Group	Quotes
Patients	“I’m not too concerned about the security. I don’t think it would be a huge risk for me even if my health information is leaked. An old saying goes, ‘the more serious the illness, the more you should tell people’.” “Because of my age, I’m not too interested in looking for new ways to do things and learn new things. But I’m not incapable of doing all that. If it’s something that must be done, I’d go through it, even if it’s a little burdensome.”
Physicians	“Considering the situation in Korea, doctor shopping [the practice of visiting different hospitals to get a diagnosis because they don’t trust a doctor’s diagnosis for their pathological symptoms] would happen more commonly because of the convenient HIE system.” “Being unable to revise the data would be a problem. We are human, and everyone makes mistakes when generating data. But there’s no opportunity to fix errors. If the transaction is made again to fix the error, patients would know that their records contain errors, which would cause conflicts between the patient and the hospital. In ophthalmology, the records of left and right eyes are switched from time to time.” “If anybody can see the treatment history, even small details would be available for lawyers to see and there’s a possibility of being dragged into conflict for everything. The ideal case would be when data are used for positive purposes such as research or health management, but health care professionals would actually become very defensive when diagnosing patients and making care plans.” “Some patients might refuse to provide data that are absolutely necessary, and it might become even harder when the physicians exchange patient information.”
Developers	“If consent is given through apps, there is a risk of health data being leaked when a mobile phone is lost or an app is hacked. In cryptocurrency systems, people cannot withdraw their money if their personal key or mobile phone is lost. The fact that the patient cannot find their own health information should be taken very seriously.” “Blockchain technology has not been around for long and is still being researched, there are still questions about the reliability of blockchain technology. In order to gain reliability of this technology, small-scale projects for building clinical study platforms should be conducted more and carried out up to actual demonstration.” “If data are lost due to carelessness or inexperience, restoring the lost data would be difficult. Health data can be disclosed if a mobile phone is lost or an app is hacked.”

### Suggestion: Function

Most patients wished they could control the information of their parents or children rather than their own information. They also suggested that health care professionals could have access to their information without their consent in an emergency. They further suggested saving rare blood type, allergies, and organ donor information on the system and sharing such information when needed. Physicians anticipated that recruiting patients for clinical trials would become easier. All 7 physicians who were interviewed had difficulties with recruiting patients for clinical trials. Even when patients are recruited through advertisement, statistical distribution cannot be taken into consideration. They regarded blockchain as a solution for solving this problem to some extent. At the same time, however, they were not comfortable with having access to too much information. They responded that the technology needs to consider how it will show only the information that physicians actually need. Developers were concerned with data storage. An extensive amount of data cannot be stored on blockchain, which may cause a significant drop in the speed. All of the developers agreed that it is appropriate for actual data to be stored at each institution, whereas data generation, search, and exchange should be available through blockchain (Table 7).

**Table 7:** Suggested functions of a blockchain-based hospital information exchange (HIE) system.

Group	Quotes
Patients	“Those who really need this service are my parents and they have dementia. It may not always be the right decision to give authorization for sharing information to oneself. Sometimes it’s better to ask for consent from the guardian.” “My elderly patients are scared of visiting hospitals alone. It’ll be great if I have the authorization to control my parents’ information and I won’t have to visit the hospital as often.” “Information on rare blood type, allergies, and organ donors should be stored and shared whenever needed.” “I wanted to participate in clinical trials, but no information was available. I wish someone would tell me.” “Technically I get to control my own information, but doctors should be able to look up my information in an emergency. If I get into an accident overseas and become unconscious, it’ll be helpful if my guardian or doctors could see my information.”
Physicians	“Recruiting clinical trial participants is very difficult, and I think that will be most useful when using blockchain in health care. Recruiting participants appropriate for each stage and conducting follow up are the hardest parts.” “Currently, recruiting patients for clinical trials is done by rule of thumb. Gender or age distribution is not considered most of the time. It’ll be much more convenient and effective if we can recruit participants by looking up patient information using blockchain.” “Because we get to see patients very briefly, there has to be a way of clearly showing only the information we need. For example, highlighting only the abnormal values or generating graphs of data for which the changes need observation.”
Developers	“Patient data will be generated from so many places. Who will be responsible for managing such a vast amount of data? It’ll be most convenient if the data are stored in the central location at the national level, but it’s not possible for the central government to manage PGHD [patient-generated health data]. It’s more efficient to store metadata in the central location, and the data itself should be saved on users’ mobile phone or other storage media.” “I think the data should be distributed for storage, and a system that manages the history data in the central location to enable searching is needed.”

### Institutions to Which Participants Are Willing to Provide Information

The interviewees were asked for what purpose and to which institutions they would be willing to provide their information. All patients were willing to provide their information to hospitals for research purposes. They did not particularly want to be compensated for providing data, but they wished to get the results if they participate in clinical trials. Among the 7 patient interviewees, only 2 were willing to provide data to profit-based corporations. Some physicians were willing to provide data to all institutions, only for public interest, or to places that give greater compensation. Most of the developers responded that they would provide information to all institutions for appropriate compensation.

## Discussion

### Principal Findings

This study was conducted in an IT-friendly tertiary hospital equipped with a government-led nationwide HIE system, and we analyzed the various expectations and concerns that doctors, patients, and developers already exposed to HIE and a PHR environment had about a potential patient-oriented blockchain-based HIE platform. This study is the first to analyze three different perspectives by interviewing all three stakeholder groups that will actually develop and use a patient-centered blockchain-based HIE.

Regarding the leakage of health information, the patient group did not have concerns, whereas the physician and developer groups expressed such concerns. We actually expected patients to be sensitive with regard to the security of their health information; however, in contrast to these expectations, they were not particularly concerned. Normally, patients attending tertiary hospitals tend to trust hospitals and doctors in South Korea. This may the reason for the lack of concern of privacy issues found in this study. Another reason is that there have been few accidents of health care information leakage so far in South Korea. By contrast, physicians and developers consider the security of health information as one of the greatest values because they directly manage a hospital information system and understand how vulnerable the system is to cyberattack. Patients are unaware of the risks resulting from the leakage of health information, whereas those who handle medical records often are more aware of the risks of leakage, thus creating a discrepancy between the patient group and physician group.

Moreover, physicians were especially concerned about the fact that errors in the data cannot be easily fixed due to the nature of blockchain technology. Instead, using blockchain technology, when medical records need to be revised, a letter of explanation must be completed and approved after which relevant departments that may be affected by the revision have to be notified (at most medical institutions). Accordingly, the revision history is uploaded to the blockchain network. However, with respect to legal conflicts arising due to the sharing of data, only the metadata of health data are stored on the blockchain network while the actual data are rarely carried by the blockchain due to speed and size issues, thus preventing all of the data from being accessed by legal personnel. The hospitals are currently obligated to provide any requested data if patients want to take legal actions against health care professionals, which indicates that the issue of rising legal conflicts will not become worse. Developers were especially concerned with the difficulty in bringing all institutions and corporations together to agree on data standardization if they were to participate in creating a network, and in actually proceeding with the standardization process. The most ideal case would be to have the central government manage all of the data; however, the study participants agreed that sharing metadata on the blockchain network and storing the actual data in the storage media of each institution or corporation are the most suitable methods, since concentration or abuse of power may occur from having exclusive rights over the data if all data are managed by a single entity. Data standardization is necessary to facilitate data exchange between different institutions. Terminology mapping must be performed for data standardization; however, this may entail an extensive amount of time and costs if detailed understanding of the business is lacking.

Patients have been frequently exchanging their health information for a variety of purposes. They were very positive about having control of their own information, with advantages of not having to visit the hospital to receive documents that need to be submitted to different hospitals, insurance companies, or other health management institutions (eg, chiropractic office, fitness center), and could avoid repeated examinations. However, most patients were afraid of and reluctant to accepting and learning new technology by themselves. They also have high trust in physicians and the guidance provided by the hospital. Most patients responded that they would like to start using new services if their doctors explain the need for using the new service while the hospital provides guidance. Furthermore, they expected doctors to be able to look up their information without their consent in emergencies, and to have control over the health information of their elderly parents or underage children. They were not against the idea of providing information for the purpose of participating in clinical research, and they wished to be provided with the results instead of compensation for providing the data. In contrast to our expectation, the patients were not particularly concerned with the leakage of their health information; because most of them lived in a different city from their elderly parents, they prefer being able to share their parents’ health information using a mobile phone, instead of having to visit the hospital every time with their parents simply to explain their conditions or to receive necessary documents for insurance claims.

Regarding this issue, all physicians agreed that the results of clinical research should be shared with the participants; however, some disagreed as to what extent the details should be shared. Some argued that it is the right of the participants to be provided with the complete results of clinical research, whereas others suggested maintaining the passive stance in which information is provided only when requested by the participant. Some physicians explained that everyone needs to agree on when and to what extent they should share research results in addition to the results of the examinations conducted when participating in clinical research. The results cannot be shared until the findings are officially approved and conclusions are made; hence, sharing results is meaningless, as argued by some physicians.

Regarding service utilization, the patients were willing to use the new service if recommended by physicians and supported by the guidance and instructions from the hospital. Physicians emphasized the importance of the government’s role in establishing an initial system and the verification process. The government should play an active role in establishing a compensation and incentive policy, provide a subsidy when building computer power—which is required for system maintenance—and issuing coins for forming an initial ecosystem.

### Blockchain and Patient-Centered HIE

According to the results of this study, all three stakeholders agreed that HIE is needed. From the physicians’ perspective, the blockchain-based patient-centered HIE is not a problem of technology. The main driver is the government’s policy to implement the system. For instance, the Japanese government is already trying to implement and evaluate several new ITs, including blockchains, to actively explore innovative and disruptive technologies through the Cabinet Secretariat’s regulatory sandbox system [51]. Further, from the developers’ perspective, there is no problem with respect to the blockchain technology itself. Rather, the problem lies in what type of information can be managed by the system. Due to the Personal Information Protection Act such as the EU General Data Protection Regulation (GDPR), Organisation for Economic Co-operation and Development (OECD) Privacy Guidelines, and Health Insurance Portability and Accountability Act (HIPAA) Privacy Rules in the United States, people can request their own data to be deleted. This is possible when the recording itself is not stored on the blockchain [52]. Therefore, it is necessary to determine the extent of the data recorded on the blockchain, such as the hash value of the recorded health data, which cannot be linked to personal information, or data that will not be a risk even if accessed. Moreover, the challenge is to justify the implementation of the blockchain in HIE. If there are incentives for data tampering and using a trusted third party is expensive, using blockchain is a reasonable solution [53,54]. Health care blockchain is not suitable for the current health care environment given the low HIE adoption rate. However, in the future, HIE will play a similar role as the internet does today, and thus HIE is expected to be used daily. In this environment, blockchain can be a powerful driver for ensuring information privacy and security.

### Limitations and Future Research

There are several limitations of this study. First, 21 participants cannot represent the opinion of the entire population, and the three groups of stakeholders cannot represent all stakeholders. Second, the age of the patient group was between 40 and 60 years, which could introduce bias in the responses. Third, patients and physicians were recruited from a single medical institution as the interviewees, thus failing to secure diversity in samples. More studies should be conducted in the future regarding these limitations. Finally, physicians and patients did not have technical knowledge of blockchain. However, we tried to provide proper background knowledge about blockchain before each interview so that the results of the interview were not biased.

### Conclusion

We analyzed the opinions of stakeholders regarding a blockchain-based, patient-centered HIE platform based on interviews with physicians, IT developers, and patients using the PARiHS framework. Most of the participants were positive about the idea of patients having the control of sharing their own health information, but were skeptical about the cooperation among various institutions and the implementation for data standardization in the establishment process, in addition to actual measures for utilization of the service. Taking these factors into consideration during planning, development, and operation of a platform will contribute to establishing practical treatment plans and tracking in a more convenient manner for both patients and physicians, and will help to expand the related research and health management industry.

## Appendix

Multimedia Appendix 1 List of interview questions.

Multimedia Appendix 2 Detailed responses to interview questions in each stakeholder group.

## Abbreviations

COREQ: Consolidated Criteria for Reporting Qualitative research
EHR: electronic health record
HIE: health Information exchange
HITECH: Health Information Technology for Economic and Clinical Health
IT: information technology
PARiHS: Promoting Action on Research Implementation in the Health Service
PHR: personal health record
