# Supplementary material for: Perspectives of Patients, Health Care Professionals, and Developers Toward Blockchain-Based Health Information Exchange: Qualitative Study
Source: J Med Internet Res. 2020 Nov 13;22(11):e18582. doi: 10.2196/18582 (PMC7695529; doi:10.2196/18582)
Supplement: Multimedia Appendix 1 [file jmir_v22i11e18582_app1.docx]

Interview Questions

| Category | Subject | Key Theme | Questions |
| --- | --- | --- | --- |
| Evidence | Patient  Physician  Developer | Awareness | - Are you aware of the PHR application provided by the hospital?  - Are you aware of the health information exchange system between hospitals?  - Are you aware of the blockchain-based health information exchange? |
|  | Physician  Developer | Awareness | - Are you aware of the patient-centered health information exchange? |
|  | Patient | Prior Experience | - Did you ever have to share your health records?  - How did you share your health records? |
|  | Physician | Prior Experience | - Have you ever used a health information exchange system before?  - Have you ever used a patient’s PGHD (Patient Generated Health Data) record for treatment?  - Have you ever recruited patients for clinical research? |
|  | Developer | Prior Experience | - Did you participate in the development of a health information exchange system?  - Did you participate in the development of the PHR application? |
| Context | Patient | Existing Problems | - Why did you choose that method?  - Were there any inconveniences? |
|  | Physician | Existing Problems | - Did you experience any inconvenience when exchanging treatment information?  - Did you experience any inconvenience when using patients’ PGHD records?  - Are there any difficulties in recruiting patients for clinical trials? |
|  | Developer | Existing Problems | - Were there any difficulties in developing the health information exchange system?  - Were there any difficulties in developing the PHR application? |
|  | Physician | Subject Population | - Which area do you practice in and how many years of experience do you have?  - How well do you think you handle the current EMR system?  - How often do you get/make referrals with HIE system in a month (in average)? |
|  | Patient | Subject Population | - Do you visit the hospital often?  - Are you interested in taking care of your health?  - Are you interested in new IT technologies?  - Do you easily adopt new technologies? |
|  | Patient | Emphasis  on Satisfaction | - Why did you decide to still use that method? |
| Facilitation | Patient | Attitudes | - What do you think of the PHR application and health information exchange service?  - Are you willing to use such a service in the future?  - What do you think of having the ownership of your health records and the power to share only certain information to an institution when necessary based on your judgment of blockchain technology? |
|  | Physician  Developer | Attitudes | - What do you think of sharing patients’ PGHD and health information together?  - What do you think of the blockchain-based patient-centered health information exchange?  - Patients can provide their health information to medical institutions as well as non-medical institutions through the blockchain-based patient-centered health information exchange. What is your opinion on this?  - Patient-centered health information exchange may lead to the formation of patient-centered health data market. What is your opinion on this? |
|  | Patient | Perceived Risk | - When using existing services, what risks do you think it entails? |
|  | Patient  Physician  Developer | Perceived Risk | - If sharing personal health records using blockchain becomes possible, what risks do you think it may entail? |
|  | Patient  Physician  Developer | Perceived Advantage | - What advantage do you think the blockchain-based health information exchange has over the information exchange service, PHR application, and HIE that are currently offered? |
|  | Patient | Suggestions | - What features do you think are absolutely necessary when planning a blockchain-based sharing service in which the user owns his or her health records and shares it with an institution when requested, only with his or her consent? |
|  | Physician  Developer | Suggestions | - Do you have any opinion on the utilization of the blockchain-based patient-centered health information exchange? |
|  | Physician | Suggestions | - Among various types of health information, which information will be helpful for treatment?  - Which type of diseases would benefit from such a system? |
|  | Developer | Suggestion | - Do you have any suggestions for improving the efficiency of developing a blockchain-based personal health record sharing system?  - Do you have any suggestions for data storage and management methods when developing a blockchain-based personal health record sharing system?  - Is there a scenario where a blockchain-based personal health record sharing system can be effectively used? |
|  | Patient | Other | - To which institutions are you willing to provide your information? |
| PHR: Personal Health Record  HIE: Health Information Exchange  PGHD: Patient Generated Health Data | | | |
