# Supplementary material for: Perspectives of Patients, Health Care Professionals, and Developers Toward Blockchain-Based Health Information Exchange: Qualitative Study
Source: J Med Internet Res. 2020 Nov 13;22(11):e18582. doi: 10.2196/18582 (PMC7695529; doi:10.2196/18582)
Supplement: Multimedia Appendix 2 [file jmir_v22i11e18582_app2.docx]

### **Appendix 2.** Responses to each question according to subgroup.

| **Key Theme** | **Questions** | **Answer** | **Patient** | **Physician** | **Developer** |
| --- | --- | --- | --- | --- | --- |
| **Evidence** |  |  |  |  |  |
| **Awareness** | Are you aware of the PHR app provided by the hospital? | Yes | 1 | 7 | 7 |
|  |  | No | 6 | 0 | 0 |
| **Awareness** | Are you aware of the health information exchange system between hospitals? | Yes | 5 | 7 | 7 |
|  |  | No | 2 | 0 | 0 |
| **Awareness** | Are you aware of blockchain-based health information exchange? | Yes | 0 | 7 | 7 |
|  |  | No | 7 | 0 | 0 |
| **Awareness** | Are you aware of patient-centered health information exchange? | Yes | - | 4 | 7 |
|  |  | No | - | 3 | 0 |
| **Experience** | Did you ever have to share your health records? | Yes | 7 | - | - |
|  |  | No | 0 | - | - |
| **Experience** | How did you share your health records? | Visit to the hospital | 7 | - | - |
| **Experience** | Why did you choose that method? | guided like that | 7 | - | - |
| **Experience** | Have you ever used a health information exchange system before? | Yes | - | 7 | - |
|  |  | No | - | 0 | - |
| **Experience** | Have you ever used a patient’s PGHD for treatment? | Yes | - | 1 | - |
|  |  | No | - | 6 | - |
| **Experience** | Have you ever recruited patients for clinical research? | Yes | - | 7 | - |
|  |  | No | - | 0 | - |
| **Experience** | Did you participate in the development of a health information exchange system? | Yes | - | - | 7 |
|  |  | No | - | - | 0 |
| **Experience** | Did you experience any inconvenience when exchanging treatment information? | Varied information, including unnecessary/inappropriate information |  |  |  |
|  |  | lack of participation | - | 2 | - |
|  |  | no answer | - | 1 | - |
| **Context** |  |  |  |  |  |
| **Problems** | Were there any inconveniences? | convenient | 0 | - | - |
|  |  | inconvenient | 7 | - | - |
| **Problems** | Why did you decide to still use that method? | because I had to | 7 | - | - |
|  |  |  |  |  |  |
| **Problems** | Did you experience any inconvenience when using patients’ PGHD records? | unavailable in EHR | - | 1 | - |
|  |  | no answer | - | 6 | - |
| **Problems** | Are there any difficulties in recruiting patients for clinical trials? | yes | - | 7 | - |
|  |  | no | - | 0 | - |
| **Problems** | Were there any difficulties in developing the health information exchange system? | poor participation | - |  | - |
|  |  | system maintenance | - |  | - |
| **Population** | How well do you think you handle the current EMR system? | Use well | - | 2 | - |
|  |  | Normal | - | 5 | - |
| **Population** | How often do you get/make referrals with HIE system in a month (on average)? | get referrals | - | 21.5 | - |
|  |  | make referrals | - | 31.8 | - |
| **Population** | Do you visit the hospital often? | Regular checkups | 5 | - | - |
|  |  | When sick, go to the hospital right away | 2 | - | - |
| **Population** | Are you interested in taking care of your health? | Exercise | 3 | - | - |
|  |  | Diet | 2 | - | - |
|  |  | Nutrients | 3 | - | - |
|  |  | nothing special | 2 | - | - |
| **Population** | Are you interested in new IT technologies? | No, it’s difficult | 6 | - | - |
|  |  | Normal | 1 | - | - |
| **Population** | Do you easily adopt new technologies? | No | 6 | - | - |
|  |  | Normal | 1 | - | - |
| **Attitudes** | What do you think of the PHR app and health information exchange service? | Sounds convenient, I’m going to use it. | 4 | - | - |
|  |  | Sounds convenient, but I’m not going to use it | 3 | - | - |
| **Attitudes** | Are you willing to use such a service in the future? | Already using | 1 | - | - |
|  |  | Only if someone can help me | 4 | - | - |
|  |  | May not use | 2 | - | - |
| **Attitudes** | What do you think of having the ownership of your health records and the right to share it with blockchain technology? | Good | 7 | - | - |
|  |  | Bad | 0 | - | - |
| **Facilitation** |  |  |  |  |  |
| **Attitudes** | What do you think of sharing patients’ PGHD and health information together? | Positive | - | 6 | 7 |
|  |  | Negative | - | 1 | - |
| **Attitudes** | What do you think of the blockchain-based patient-centered HIE? | Positive | - | 4 | - |
|  |  | Negative | - | 3 | - |
| **Attitudes** | Patients can provide their health information to medical institutions as well as nonmedical institutions through the blockchain-based patient-centered health information exchange. What is your opinion on this? | Positive | - |  | 6 |
|  |  | Negative | - | - | 1 |
| **Attitudes** | Patient-centered health information exchange may lead to the formation of patient-centered health data market. What is your opinion on this? | Positive | - | 3 | 7 |
|  |  | Negative | - | 4 | - |
| **Perceived Risk** | When using existing services, what risks do you think it entails? | nothing special | 4 | - | - |
|  |  | security | 3 | - | - |
| **Perceived Risk** | If sharing personal health records using blockchain becomes possible, what risks do you think it may entail? | nothing special | 2 | - | - |
|  |  | security | 4 | 5 | 4 |
|  |  | cannot be modified | - | 1 | - |
|  |  | too much information | 1 | 1 | - |
|  |  | Unreliable | - | - | 3 |
| **Perceived Advantage** | What advantage do you think the blockchain-based health information exchange has over the information exchange service, PHR app, and HIE that are currently offered? | nothing special | 3 | 5 |  |
|  |  | efficiency | 1 | - | - |
|  |  | wide range of information | 2 | - | - |
|  |  | other | 1 | 2 | - |
| **Suggestions** | Among various types of health information, which information will be helpful for treatment? | medication | - | 3 | - |
|  |  | diagnosis | - | 2 | - |
|  |  | lab result | - | 1 | - |
